# Rice Stripe Mosaic Virus, a Novel Cytorhabdovirus Infecting Rice via Leafhopper Transmission

**DOI:** 10.3389/fmicb.2016.02140

**Published:** 2017-01-04

**Authors:** Xin Yang, Jilei Huang, Chuanhe Liu, Biao Chen, Tong Zhang, Guohui Zhou

**Affiliations:** ^1^Guangdong Province Key Laboratory of Microbial Signals and Disease Control, College of Agriculture, South China Agricultural UniversityGuangdong, China; ^2^Instrumental Analysis and Research Center, South China Agricultural UniversityGuangdong, China

**Keywords:** Rice stripe mosaic virus, rice viral disease, cytorhabdovirus, leafhopper, *Recilia dorsalis*

## Abstract

A new rice viral disease exhibiting distinct symptoms—yellow stripes, mosaic and twisted tips on leaves—was found in China. Electron microscopy of infected leaf cells revealed the presence of bacilliform virions and electron-translucent granular-fibrillar viroplasm in the cytoplasm. The enveloped viral particles were 300 to 375 nm long and 45 to 55 nm wide. The leafhopper *Recilia dorsalis* was able to transmit the virus to rice seedlings, which subsequently exhibited symptoms similar to those observed in fields. The complete genome of the virus was obtained by small-RNA deep sequencing and reverse transcription-PCR product sequencing. The anti-genome contains seven open reading frames (ORFs). The deduced amino acids of ORF1, ORF5, and ORF7 are, respectively, homologous to the nucleocapsid protein (N), glycoprotein (G), and large polymerase protein (L) of known rhabdoviruses. The predicted product of ORF2 is identified as a phosphoprotein (P) based on its multiple potential phosphorylation sites and 12.6 to 21.0% amino acid (aa) identities with the P proteins of plant rhabdoviruses. The product of ORF4 is presumed to be the viral matrix (M) protein for it shares 10.3 to 14.3% aa identities with those of other rhabdoviruses. The above five products were confirmed as the viral structural proteins by SDS-PAGE and aa sequencing analyses of purified virus preparation. ORF3 and ORF6 are considered to encode two nonstructural proteins with unknown functions. Phylogenetic analysis based on protein N, G, and L amino acid sequences indicated that the isolated virus, which we have tentatively named Rice stripe mosaic virus (RSMV), is a new species in the genus *Cytorhabdovirus*. To our knowledge, RSMV is the only cytorhabdovirus naturally infecting rice and the first reported leafhopper-transmitted cytorhabdovirus. Our surveys of rice fields indicate that RSMV occurs frequently in Guangdong Province, China. Although the disease incidence is low at present, it might become serious with the vector insect population increasing.

## Introduction

Rice (*Oryza sativa*) is one of world’s major cereal food crops. In Asia, where more than 90% of rice production takes place (Bheemanahalli et al., 2016), rice viral diseases have recently had a serious effect on yield (Uehara-Ichiki et al., 2013). The International Committee on Taxonomy of Viruses^1^ (ICTV) currently recognizes 14 rice viruses, all arthropod-borne: one species each in genera *Benyvirus, Bymovirus, Nucleorhabdovirus, Oryzavirus, Sobemovirus, Tungrovirus*, and *Waikavirus*, two species in *Fijivirus*, two species in *Phytoreovirus* and three species in *Tenuivirus*. Five of them, *Rice yellow stunt nucleorhabdovirus* (RYSV, also named as Rice transitory yellow virus, RTYV, *Nucleorhabdovirus*), *Rice stripe tenuivirus* (RSV), *Rice yellow mottle sobemovirus* (RYMV), *Rice stripe necrosis benyvirus* (RSNV) and *Rice necrosis mosaic bymovirus* (RNMV) are distributed in mesophyll cells and induce yellowing or mosaic symptoms in infected leaves, while the remainders parasitize rice phloem cells and cause rice dwarfing and dark green leaves.

Rhabdoviruses, which have a negative-sense RNA genome of 11–16 kb, form a large family in the order *Mononegavirales* (Afonso et al., 2016; Dietzgen et al., 2016). This family is characterized by a broad host range including vertebrates, invertebrates, monocots and dicots, and some members are pathogens with significant impacts on public health, crop and livestock production (Jackson et al., 2005; Kuzmin et al., 2009; Dietzgen et al., 2016). In general, the genomes of rhabdoviruses encode at least five canonical proteins in the following conserved order: nucleocapsid protein (N), phosphoprotein (P), matrix protein (M), glycoprotein (G) and large polymerase protein (L) (3′-N-P-M-G-L-5′) (Jackson et al., 2005; Ammar et al., 2009; Kormelink et al., 2011). Besides, two or more accessory genes are often located in the genome between N–P, P–M, and/or G–L genes (Walker et al., 2011).

As currently circumscribed, the family *Rhabdoviridae* comprises nine genera of animal-infecting viruses (*Ephemerovirus, Lyssavirus, Novirhabdovirus, Perhabdovirus, Sigmavirus, Sprivivirus, Tibrovirus, Tupavirus*, and *Vesiculovirus*) and four plant-infecting viruses (*Cytorhabdovirus, Dichorhavirus, Nucleorhabdovirus*, and *Varicosavirus*) (Afonso et al., 2016; Dietzgen et al., 2016). In addition, more than 75 plant rhabdoviruses have not been assigned to a genus. Arthropods, including aphids, leafhoppers and planthoppers, are common vectors for plant rhabdoviruses in nature (Jackson et al., 2005; Ammar et al., 2009).

The members of genus *Nucleorhabdovirus* are mainly transmitted by leafhoppers or planthoppers, and infect monocots and dicots in nature. Currently, RYSV is only species known naturally infecting rice (Huang et al., 2003). In the genus *Cytorhabdovirus*, the species are mainly transmitted by planthoppers or aphids, and infect monocots and dicots in nature. Neither rice infection nor leafhopper transmission has been observed in any members of this genus, except *Wheat american striate mosaic cytorhabdovirus* (WASMV) which can be transmitted by a leafhopper, *Endria inimical* (Jackson et al., 2005). *Dichorhabdovirus* and *Varicosavirus* are two new genera which recently approved by the ICTV (Dietzgen et al., 2014; Afonso et al., 2016). In the genus *Dichorhabdovirus*, which includes two species, orchid fleck virus (OFV) and coffee ringspot virus (CoRSV) infect monocots and dicots, respectively, through mite transmission, and in the genus *Varicosavirus*, which contains only one species, lettuce big-vein associated virus (LBVaV) infects dicots by fungi transmission (Sasaya et al., 2002, 2004; Kondo et al., 2006; Ramalho et al., 2014).

In 2015, a new rice disease was observed in southern China. Affected rice plants exhibited slight dwarfing and the initial appearance of yellow stripes on leaves followed by mosaic and twisting of some leaves, and produced inferior heads bearing only a few, mostly unfilled grains. In this study, we detected a novel plant rhabdovirus in the infected plants by electron microscopy and small RNA sequencing. Artificial inoculation with the leafhopper *Recilia dorsalis* (Hemiptera: Cicadellidae) confirmed the novel virus as the disease pathogen. We next characterized the morphology and distribution of the virion in infected leaf cells, the viral structural proteins, its natural plant host range and insect vectors, features of the viral genome and phylogenetic relationships. We propose to name this virus as Rice stripe mosaic virus (RSMV), and classify it as a new member of the genus *Cytorhabdovirus* of the family *Rhabdoviridae*.

## Materials and Methods

### Sample Collection and Virus Transmission

Plants with distinct symptoms were collected from a rice field in Taiping, Luoding, Guangdong Province, southern China, during October 2015 to May 2016. Representative weeds (*Digitaria sanguinalis, Cynodon dactylon, Leptochloa chinensis, Eleusine indica, Paspalum distichum*, and *Monochoria vaginalis*) as well as leafhoppers (*Recilia dorsalis*) were collected from or adjacent to the diseased fields. The leafhoppers was identified according to the document by Motschulsky (1859). Insect transmission of the virus was conducted with leafhopper *R. dorsalis*. Nonviruliferous leafhoppers collected from a non-diseased field were reared over two generations on four-leaf-stage seedlings of rice cultivar Taichung Native 1. The seedlings were maintained in a plant growth chamber at 28°C and 80% relative humidity under a 16-h light/8-h dark photoperiod. The next generation nonviruliferous nymphs were then placed on diseased rice for a virus acquisition access period of 10 days. Rice seedlings at the three-leaf stage were inoculated with the viruliferous nymph leafhoppers for 3 days. The seedlings were then sprayed with insecticide (0.2% Isoprocarb) to kill all leafhoppers and were subjected to pathogen detection by electron microscope, small RNA sequencing and reverse transcription-PCR (RT-PCR) another 10 days later. Mechanical transmission of the virus was attempted with reported previously method (Lamprecht et al., 2010).

### Electron Microscopy

Crude sap from the viruliferous leafhopper inoculated rice leaves was negatively stained with 2% phosphotungstic acid (pH 6.8). Ultrathin sections were cut on an ultramicrotome and stained with uranium acetate and lead citrate (Li et al., 2015). Virion morphology and viroplasm distribution in the infected cells were examined under a transmission electron microscope (TECNAI G212, Holland).

### Virus Purification and Structural Protein Analyses

Virus purification was conducted according to Heim et al. (2008). Briefly, two hundred grams of symptomatic rice leaves (grown in greenhouse after viruliferous leafhopper inoculation) were ground in extraction buffer (100 mM Tris-HCl, pH 8.0), and filtered through cheesecloth and centrifuged at 5,000 rpm for 10 min at 4°C. The supernatant was ultracentrifuged (45,000 rpm) for 30 min at 4°C and the pellets were resuspended in 3 mL of 100 mM extraction buffer. The suspension was loaded on a discontinuous 20–40% sucrose density and centrifuged at 30,000 rpm for 2 h at 4°C. The light band was collected and ultracentrifuged (45,000 rpm) for 40 min at 4°C, the pellet was resuspended in 100 μL resuspension buffer (50 mM Tris-HCl, pH 8.0). All the ultracentrifuged procedures were done in a Beckman 100 Ti Rotor(XL-100K, Beckman, CA, USA).

After evaluated by electron microscope observation, the purified virus preparation was disrupted in loading buffer (50 mM Tris-HCl, pH 6.8, 2% SDS, 1% 2-mercaptoethanol, 10% glycerol and 0.1% bromophenol blue), then the viral structural proteins were separated on 12% SDS-PAGE (Kondo et al., 2009). The isolated protein bands were cut and digested with trypsin, peptides were sequenced by liquid chromatograph-mass spectrometry (LC-MS) (BGI, Shenzhen, China).

### Small RNA Library Preparation and Viral Genome Sequencing

Total RNA was extracted from pools of five virus-infected rice leaves using an RNeasy Plant Mini Kit (Qiagen Germany). The RNA fragments between 140 and 150 nt in size were isolated on a 12% polyacrylamide gels as describled by Wang et al. (2010), which was followed by sequencing on an Illumina Hiseq2500 sequencer performed by Sangon Biotech (Shanghai, China). The sequencing data were analyzed with SPAdes software (Sangon Biotech) to obtain contigs homologous to known viruses. To determine the nearly complete viral genome, specific primers were designed to close gaps between the three obtained contigs (a, b and c), which were, respectively, similar to rhabdovirus N, G, and L genes (**Figure 3A**). To generate the terminal sequences of the viral genome, an RNA ligase-mediated rapid amplification of cDNA end (RLM-RACE) (Liu and Gorovsky, 1993) was conducted using the viral RNA and viral cDNA after ligating their 3′ end with an adaptor (5′- PO4-ttccttatgcagctgatcactctgtgtcagttccagtcacgaca-NH2-3′) respectively. To avoid the potential mis-assembly, the obtained viral sequence was confirmed by re-sequenceing of the RT-PCR products from corresponding genomic regions. All primers used in this study are listed in Supplementary Table S1.

### Viral Genomic Sequence and Phylogenetic Analyses

The complete nucleotide sequence of the discovered virus was analyzed with Lasergene DNAStar software. Predicted amino acids were compared using the NCBI BLASTp program^2^, and potential phosphorylation and glycosylation sites were determined with NetPhos 2.0 (Blom et al., 1999) and NetNglyc1.0^3^, respectively. The nuclear localization signals was predicted by cNLS Mapper (Kosugi et al., 2009). Sequence alignments of the nucleotide and predicted amino acids of the novel and other plant rhabdoviruses were carried out in CLUSTAL W. Phylogenetic trees were generated from the aligned sequences in MEGA 5.0 using the neighbor-joining method (Tamura et al., 2011).

### Detection and Investigation of the Virus

Total RNAs were isolated from rice leaf tissues or individual insects using an RNeasy Plant Mini Kit (Qiagen). RT-PCR amplification was carried out using a One-Step RNA PCR kit (Takara, Dalian, China) and the virus-specific detection primers listed in Supplementary Table S1. The following RT-PCR conditions were used: initial steps of 50°C for 30 min and 94°C for 2 min, followed by 35 cycles of 94°C for 30 s, 55°C for 30 s and 72°C for 30 s, and a final extension of 72°C for 10 min. The amplified DNA fragments were excised from a 1.2% agarose gel after electrophoresis and sequenced directly for analysis.

Field survey was carried out in Guangdong Province, China during 2015–2016. Suspicious rice samples were collected and analyzed by RT-PCR, with virus-positive products then directly sequenced.

## Results

### Disease Symptoms

**FIGURE 1:**
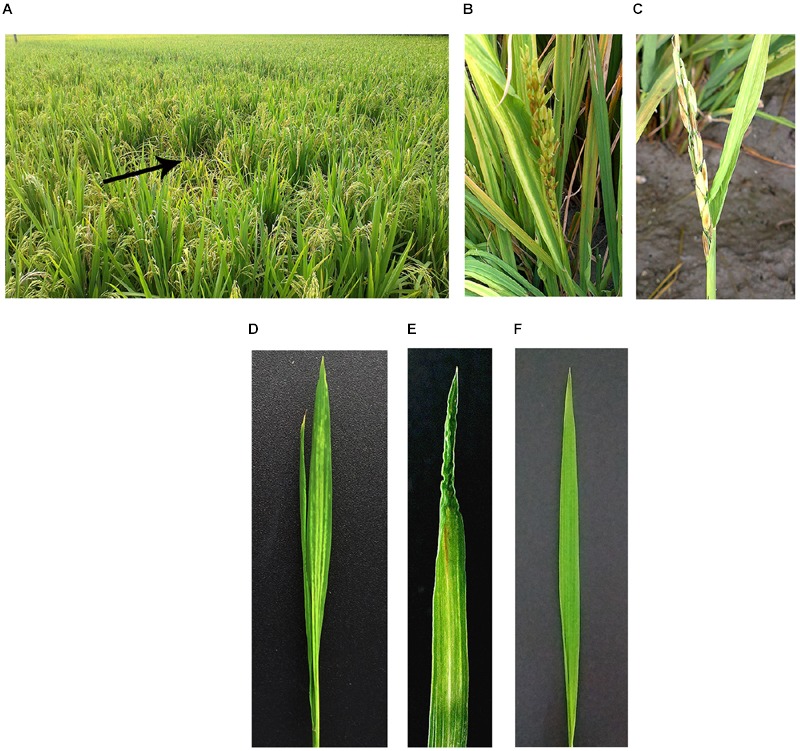
**Symptoms of Rice stripe mosaic virus (RSMV)-infected rice.** Infected rice in the field **(A–C)**. Infected rice leaves at 21 days after inoculation by the viruliferous vector *Recilia dorsalis* **(D,E)** and negative controls **(F)**. The black arrow indicates RSMV-infected rice plant whose symptoms was shown in **(B,C)**. The rice cultivar is Wuyou 736 in **(A–C)**, and Taichung Native 1 in **(D–F)**.

Our field investigation revealed that diseased plants exhibited slight dwarfing, with leaves showing yellow stripes, a mosaic appearance and occasional twisting. Diseased plants produced inferior heads that tended to remain only halfway emerged from leaf sheaths (**Figures 1A,B**). The grains were often unfilled (**Figure 1C**). Ten days after leafhopper *R. dorsalis* vector inoculation, new leaves of three-leaf-stage rice seedlings developed obvious yellow stripes, subsequently displayed mosaic symptoms and inward-curled tips (**Figures 1D,E**). While the control rice leaves with nonviruliferous leafhopper transmission showed no symptoms (**Figure 1F**).

### Virion Morphology and Cytopathology

**FIGURE 2:**
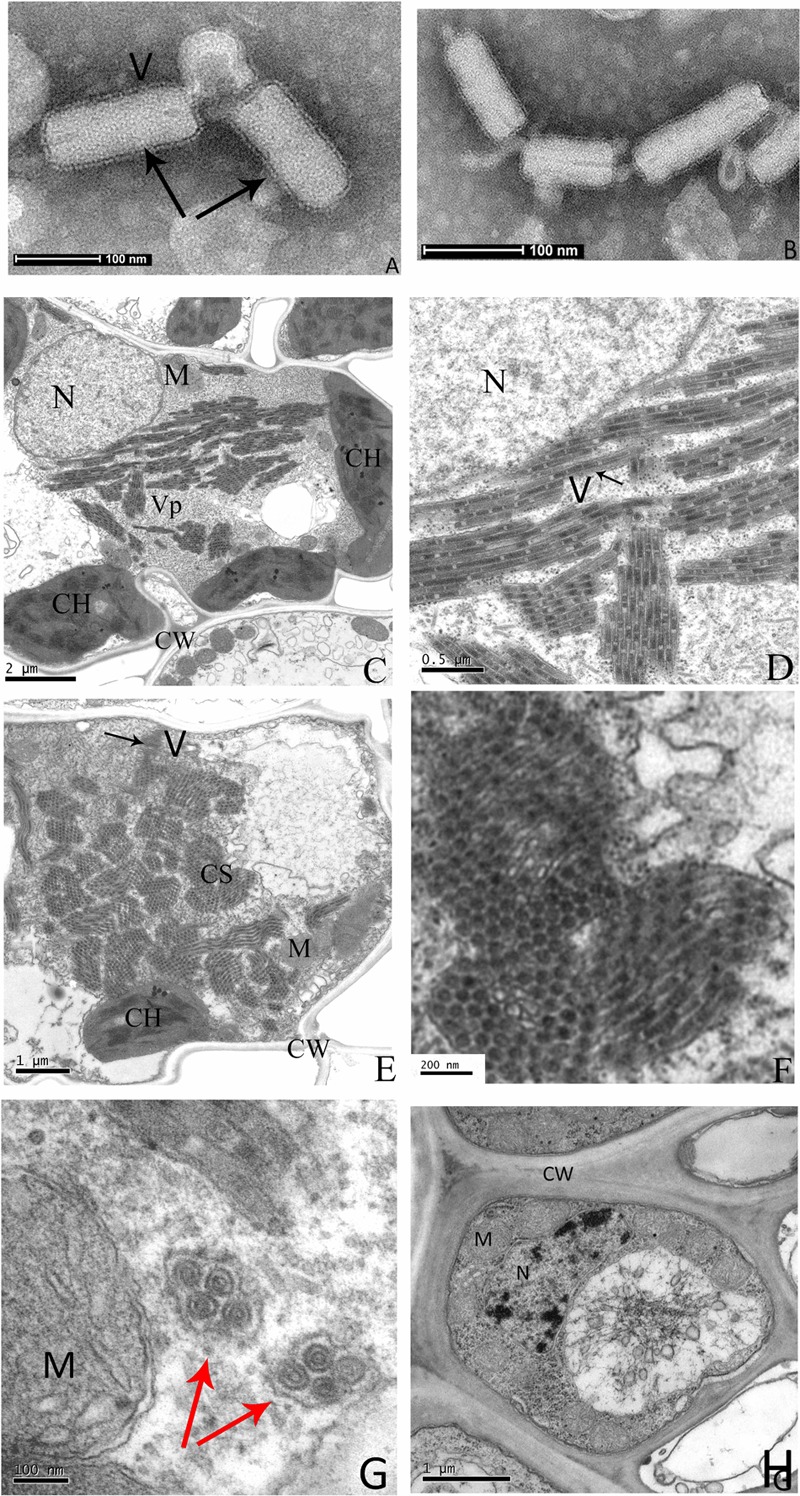
**Electron micrograph of infected rice leaves.** Negative-stained sap of infected rice leaves **(A,B)**. Ultrathin sections of infected rice leaves showing RSMV virions and cytopathological structures **(C–G)**, the enlarged CS of **(E)** is shown in **(F)**. The uninfected rice leaves cells are shown in **(H)**. Black arrows indicate the RSMV virions with enveloped, red arrows indicate the vesicle structure with gathered virions in it. CW = cell wall, CH = chloroplast, CS = crystalline structure, M = mitochondrion, N = nucleus, V = Virion, and Vp = viroplasm.

Negative staining of crude sap from diseased rice leaves revealed many enveloped bacilliform matured virions with 300–375 nm in long and 45–55 nm in wide (*n* = 50), and some broken viral particles with a minimum length 130 nm (**Figures 2A,B**). These virion sizes are similar to barley yellow striate mosaic virus (BYSMV) (Yan et al., 2015) and within the range of known members of plant rhabdoviruses (Jackson et al., 2005). These particles, which were absent from the nucleus, accumulated in cytoplasm and formed large numbers of crystalline structures that nearly occupied the entire cytoplasm space (**Figures 2C–F**). Some virions were gathered and surrounded in vesicle (**Figure 2G**). Virions were found in infected leaf and vascular system cells, but were not present in cells of healthy plants (**Figure 2H**).

### Characteristics of Complete Viral Genome and Virus Derived Amall RNA

**FIGURE 3:**
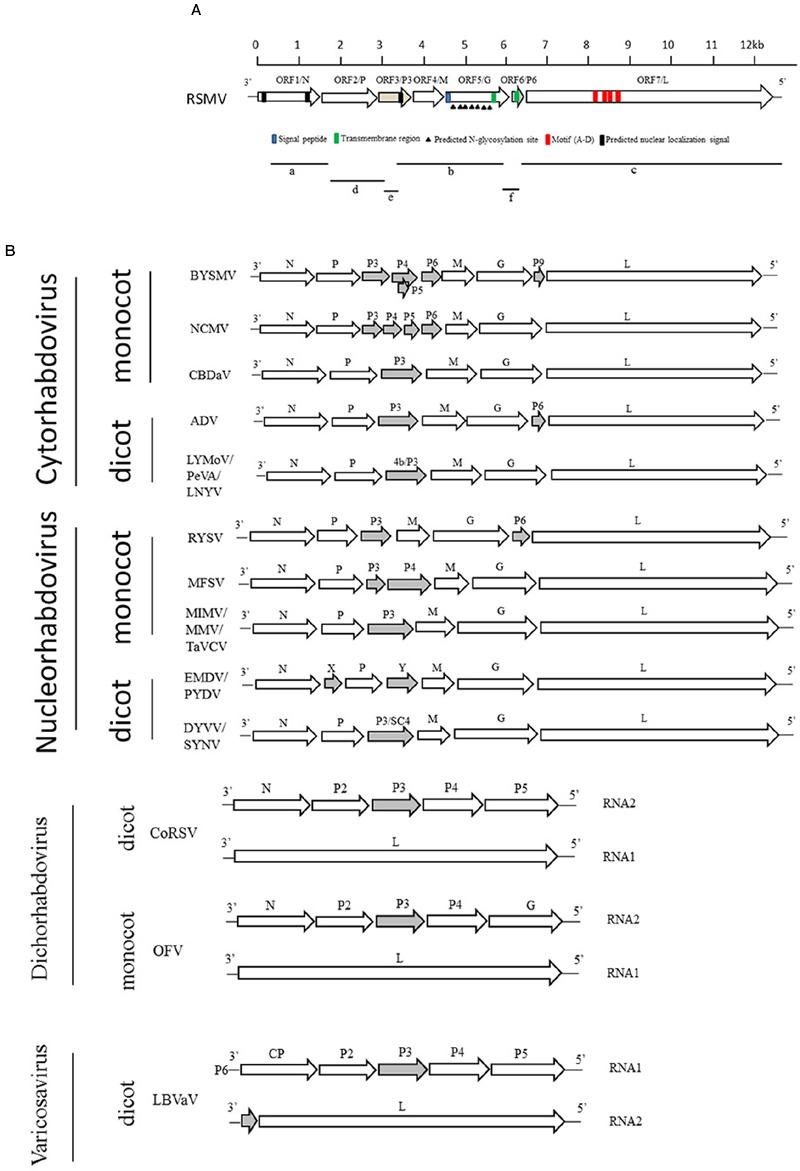
**Comparative genome organization of RSMV and plant-infecting rhabdoviruses. (A)** Putative gene of RSMV are shown as black arrows on the anti-genomic strand. Fragments a, b, and c are contigs from small RNA sequencing that are similar to rhabdovirus N, G, and L genes, respectively. Contig fragments d, e, and f showed no homology with any known viruses in NCBI and were mapped to the complete genome of RSMV. **(B)** Comparison of the genome structure of plant rhabdoviruses. Open reading frames (ORFs) for the main structural protein genes N, P, M, G, and L are indicated as open arrows. Ancillary genes are shown as gray arrows.

Approximately 10,119,447 individual small RNA raw reads were produced by deep sequencing. After removal of the adaptor sequence and low quality reads, 2,124,776 unique reads were obtained and all the small RNAs length was distribution between 17 and 35 nt. *De novo* assembly of the small RNAs by the Sangon Biotech Co., Ltd (Shanghai, China) with the SPAdes software obtained 1097 contigs. BlastN searches were performed to identify virus sequence with these contigs in the native database, 357 contigs were not matched to any viral genomes. The rest of contigs were searched again by BlastX in the National Center for Biotechnology Information (NCBI) data base. Three contigs (fragments a, b, and c in **Figure 3A**) showed significant similarity to rhabdoviral N (40% amino acid [aa] identity with BYSMV), G (23% aa identity with northern cereal mosaic virus, NCMV) and L (39% aa identity with BYSMV) proteins. To recover almost the entire genome, specific primers (Supplementary Table S1) based on the sequence of these three contigs were designed to close a few internal gaps. In addition, RT-PCR with an adaptor primer was performed to obtain viral genome terminal sequences. By comparing the three contigs against the whole genome as a reference, three other contigs were mapped to their genomic positions (fragments d, e, and f in **Figure 3A**). Finally, overlapping RT-PCR was performed and amplicons were directly sequenced from both directions to verify the obtained RSMV genome sequence. The complete RSMV genome (GenBank accession no. KX525586) was found to comprise 12,782 nucleotides (nt), with 3′ leader and 5′ trailer sequences containing 89 and 296 nt, respectively.

In the developed small RNA library, the major size of unique small RNAs is 24 nt reads (33.4%), followed by 21 nt (10.4%) (Supplementary Figure S1A), while for the RSMV-derived small RNAs, 21 nt reads is majority (13.1%) (Supplementary Figure S1B), which is similar to other virus-derived small RNAs in *Arabidopsis* (Wang et al., 2010). In addition, the 5′ termini of RSMV-derived small RNAs are mostly Uridines (U), following with Adenines (A), Cytidines (C), and Guanines (G) in order (Supplementary Figure S1C). Furthermore, the mapped viral small RNAs to RSMV genome revealed that genome-derived small RNAs are more than antigenome-derived ones, and highest peaks are located in 3072 and 1701 nucleotide position of genome and anti-genome, respectively (Supplementary Figure S1D).

## Rsmv Genome Analysis and Comparison With Other Plant Rhabdoviruses

The complementary-sense RNA of RSMV was predicted to contain seven ORFs and has similar gene arrangement to most rhabdoviruses (**Figure 3**). The features of their encoded proteins are shown in **Table 1**. ORF1, containing 1,476 nt, putatively encodes a structural protein N. Sequence identities between the N protein of RSMV and those of plant rhabdoviruses (*Cytorhabdovirus, Dichorhavirus, Nucleorhabdovirus* and *Varicosavirus*) range from 32.7 to 50.8% (nt) and 11.6 to 33.0% (aa). The aa sequence of the RSMV N protein putatively contains two nuclear localization signals (NLSs) at amino (aa positions 14–45) and carboxy (aa positions 440–474) termini (**Figure 3A**). ORF2 is composed of 1,128 nt. Sequence identities between the encoded protein of RSMV and P proteins of plant rhabdoviruses range from 34.8 to 49.0% (nt) and 12.6 to 21.0% (aa). Although these aa identities are low, the ORF2-encoded protein is acidic (isoelectric point [pI] = 4.96) and possesses potential phosphorylation sites, similar to other rhabdoviruses (Jackson et al., 2005). These characteristics suggest that ORF2 encodes the viral structural protein P. ORF3, comprising 534 nt, has sequence identities with non-structural protein P3 of plant rhabdoviruses ranging from 27.6 to 43.7% (nt) and 10.8 to 19.7% (aa). It is a basic protein (pI = 9.56), contains a NLS in the carboxy terminus (aa positions 143–173), and may be function as a movement protein. ORF4, containing 525 nt, was predicted to encode an matrix (M) protein (pI = 5.54). Sequence identities between the ORF4-encoded protein and plant rhabdoviruses range from 25.2 to 37.2% (nt) and 9.3 to 15.2% (aa). ORF5, which contains 1,611 nt, was predicted to encode a structural protein G having a sequence identity with G proteins of plant rhabdoviruses ranging from 36.0 to 49.6% (nt) and 11.0 to 22.5% (aa). The amino terminal (aa positions 1–19) of this putative RSMV G protein possesses a signal peptide and seven potential glycosylation sites (aa positions 65, 232, 265, 366, 381, 403, and 454). In addition, a transmembrane domain was predicted in the carboxy terminal (aa positions 481–503) (**Figure 3A**). ORF6 contains 201 nt and putatively encodes a basic ancillary protein (pI = 9.9) of unknown function, which we designated as P6. P6 contains a transmembrane domain (aa positions 26–43). Sequence identities between P6 and proteins of ADV, BYSMV and RYSV range from 41.5 to 48.4% (nt) and 9.8 to 34.3% (aa). The 6,201-nt ORF7 putatively encodes an L protein containing motifs characteristic of RNA-dependent RNA polymerases of negative-strand RNA viruses (**Figure 3A**) and including the GDNQ motif thought to represent the catalytic center (Dietzgen et al., 2006). Sequence identities between this L protein and those of other plant rhabdoviruses range from 39.4 to 52.5% (nt) and 20.9 to 39.4% (aa). Sequence identities (nt and aa) of these RSMV proteins compared with those of plant rhabdoviruses are listed in Supplementary Table S2.

**Table 1:** Features of proteins encoded in the positive-sense orientation by the Rice stripe mosaic virus (RSMV) anti-genome.

Gene name	Open reading frames (ORF) position	Calculated *Mr* (KDa)	Isoelectric point (pI)	BlastP match in NCBI	*E*-value	Query coverage (%)	Identities with plant rhabdoviruses (%)		No. of potential phosphorylation sites	
							Nucleotide	Amino acid	Ser	Thr	Tyr
ORF1/N	90–1565	54.4	8.68	Nucleoprotein (BYSMV)	2.00E-55	93	32.7–50.8	11.6–33.0	25	14	7
ORF2/P	1677–2804	41.9	4.96	No significant	None	None	34.8–49.0	12.6–21.0	29	4	6
ORF3/P3	3014–3547	20.1	9.56	No significant	None	None	27.6–43.7	10.8–19.7	9	2	1
ORF4/M	3738–4262	19.6	5.54	No significant	None	None	25.2–37.2	9.3–15.2	5	4	3
ORF5/G	4384–5994	60.1	5.76	Glycoprotein (NCMV)	7.00E-26	93	36.0–49.6	11.0–22.5	20	13	6
ORF6/P6	6013–6213	8.0	9.9	No significant	None	None	41.5–48.4	9.8–34.3	1	0	0
ORF7/L	6286–12486	235.9	7.72	L protein (BYSMV)	0	99	39.4–52.5	20.9–39.4	74	25	20

Junctions between these protein-encoding genes identified in RSMV were analyzed with CLUSTA W. These sequences share three conserved regions: gene end, intergenic sequence and gene start (**Table 2**), which is a common characteristic of other rhabdoviruses (Yan et al., 2015; Dietzgen et al., 2006). The gene end (3′-AUUCUUUUU-5′) is similar to those of other plant rhabdoviruses. The first nucleotide (G) of the intergenic sequence is highly conserved in all reported plant rhabdoviruses. The gene start sequence of RSMV is predictively identical to that of known cytorhabdoviruses or varicosaviruses, which is 3′-CU-5′ (**Table 2**).

**Table 2:** Gene junction regions of RSMV and other rhabdoviruses.

	RSMV	Gene end (3′)	Intergenic sequence	Gene start (5′)
	3′le/N	AUUGUUUUCUUUUU	GCU	CUG
	N/P	AUUCUUUUU	GCU	CUG
	P/M	AUUCUUUUU	GCU	CUG
	M/P4	AUUCUUUUU	GCU	CUG
	P4/G	AUUCUUUUU	GCU	CUG
	G/P6	AUUUUUU	GUCU	CUG
	P6/L	AUUCUUUUU	GCU	CUG
	L/5′tr	CUUUUU	GCU	CUG
	Consensus	AUUCUUUUU	GCU	CUG

Cytorhabdovirus	ADV	CCAAAUUAUUU	GAU	CUU
	LNYV	AUUCUUUU	G(N)_n_	CUU
	NCMV	AUUCUUUUU	GACU	CUA
	BYSMV	AUUAUUUUU	GA	CUC
	LYMoV	AUUCUUUU	G(N)_n_	CUN

Nucleorhabdovirus	DYVV	AUUCUUUUU	GGU	UGU
	PYDV	AUUAUUUUU	GGG	UUG
	RYSV	AUUCUUUUU	GGG	UUG
	SYNV	AUUCUUUUU	GG	UUG
	MFSV	UUUAUUUU	GUAG	UUG
	EMDV	AAUUAUUUUU	GGG	UUG
	MIMV	AAUUCUUUUU	GGG	UUU/G
	MMV	AAUUCUUUUU	GGG	UUG/A
	TaVCV	AAUUCUUUUU	GGG	UUG

Dichorhabdovirus	CoRSV	UAAAUUUAUUUU	GUA	GUU
	OFV	UAAAUUUA/CUUUU	GU	UUG/N

Varicosavirus	LBVaV	AUAAUCUUUUUU	G	CUCU

Our analysis revealed that RSMV 3′ leader and 5′ trailer sequences include a short complementary section that can putatively form a panhandle structure, a feature common to other rhabdoviruses (Supplementary Figure S2). In all known cytorhabdoviruses, these complementary sequences start with UGC/ACG (except for a G/C in the end of 3′ end of colocasia bobone disease-associated virus, CBDaV); interestingly, in RSMV they begin with UUC/AAG—in other words, the second complementary nucleotide is U/A, not G/C.

### Confirmation of Viral Structural Proteins

**FIGURE 4:**
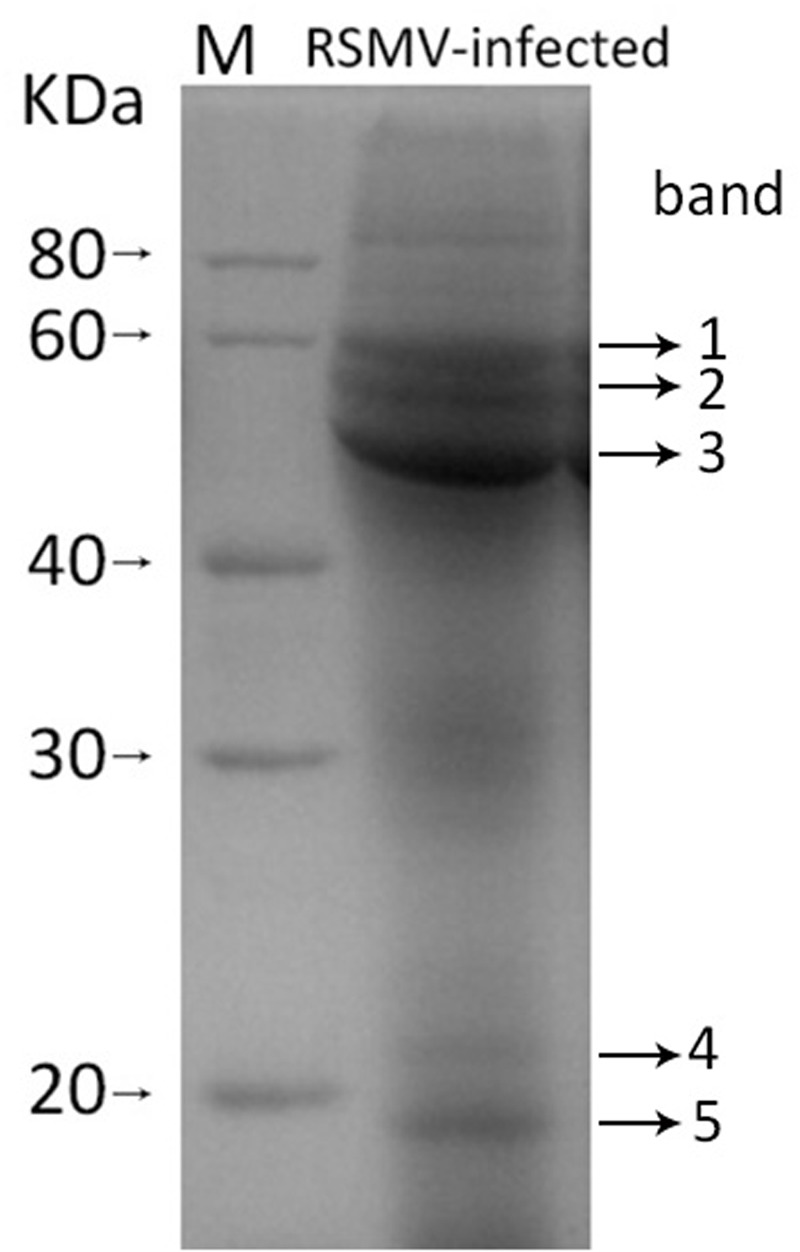
**SDS-PAGE pattern of purified SMRV preparation.** The sample was resolved in 12% SDS-PAGE gel followed by staining with Coomassie brilliant blue-R250. Five protein bands (band 1 to 5) were analysis by LC-MS.

To confirm the viral structural proteins predicted by sequence identity, purified virus preparations were disrupted and the proteins were isolated by SDS-PAGE. Five protein bands with approximately molecular weight of 60, 55, 43, 21, and 19 KDa were displayed (**Figure 4**). Based on their size, band 1, 2, 3, and 5 probably correspond the predicted protein G, N, P, and M, respectively, while band 4 lacked counterpart. These bands were cut out and digested with trypsin, and then the peptides were sequenced by LC-MS. 57, 58, 96, 21, and 72 unique aa sequences were obtained from band 1, 2, 3, 4, and 5, respectively. Among them, three peptides from band 1 were successfully mapped on to the predicted RSMV protein G, 27 from band 2 on to the protein N, two from band 3 on to the protein P, and six from band 5 on to the M, but none from band 4 on to any predicted viral protein (**Table 3**). Other peptide sequences were matched with rice, bacteria or unknown proteins. Therefore, we concluded that RSMV encoded G, N, P, M, and L (too large to isolate by SDS-PAGE) are the viral structural proteins which accord with the prediction by homology analyses.

**Table 3:** The identified peptides sequences of purified virus preparations by LC-MS analysis.

Sample	No. of identified peptides	RSMV protein	No. of peptides mapped on to viral protein	Identified peptides sequences
Band 1	57	G protein	3	EIGNTFQVGHVIEPSSMIEVTDPLNVR; GSEEAEIVDSVNK; TAQIVLDR
Band 2	58	N protein	27	AAYEAIGDEDSK; AEGVRDPMNIAGLDDISSR; AFLDDEEDDTAK; ASAGEYSSAMK; ASDPDVSDVTEVK; SEYELTR; FGPFLAAYLMR; ATANTAWIAAQEAQK; ATLDSATTDADTK; DALDAIMDILISFESTTK; DLGNSSPSTYAVER; DPMNIAGLDDISSR; GSSNFFLTDYLPK; IASNVTESWEHMK; IILAEAPK; IVSTQFFQSLQTK; KAAYEAIGDEDSK; LSLVPENTK; KGSSNFFLTDYLPK; LDQSTAEAGILR; LLVLASHLYETK; LTSNYLIGAMR; LYSISPEAYSDDK; LYSISPEAYSDDKFDK; NFYGYDAPSDLNCPEAGFLEQLK; SLFGDAGETVTDESK; YVAVLPLAYSGMHAMK
Band 3	96	P2 protein	2	AVISDSELNQAIR; IPGEIVSAVK
Band 4	21	None	0	–
Band 5	72	P4 protein	6	DIYYLEVSNK; MSVTLIMEMNEDTPLK; TLYITGGGTSFR; VINEETANQFIINDNAVR; VTGGSQLDYTSLIGSK; YPSYNAFESIFK

### Phylogenetic Relationships between RSMV and Known Plant Rhabdoviruses

**FIGURE 5:**
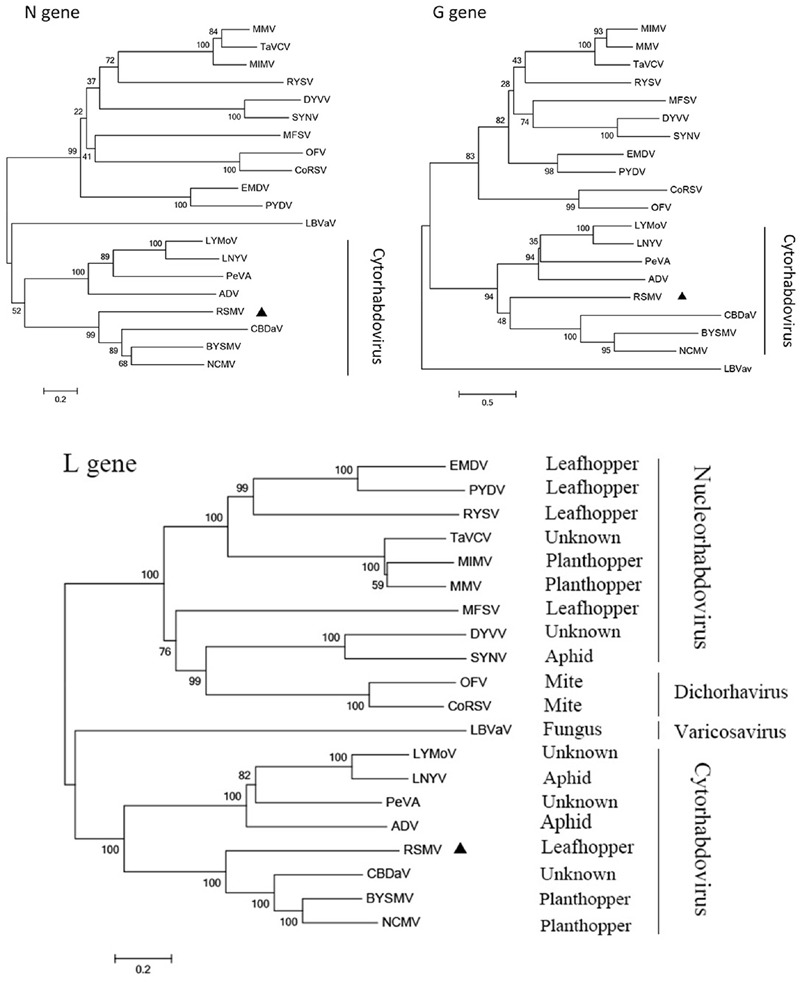
**Molecular phylogenetic tree of N, G, and L genes of RSMV.** The phylogenetic tree was constructed from N, G, and L gene sequences by neighbor-joining method based on poisson model. Bootstrap support (%) based on 1,000 bootstrap replicates is shown above and below nodes. RSMV is labeled with a black triangle.

To reveal the relationship of RSMV to other rhabdoviruses, we constructed phylogenetic trees of N, G and L protein (aa) sequences. According to the generated trees, RSMV appears to be a new member of cytorhabdovirus and is most closely related to CBDaV (**Figure 5**), and the genome organization is similarity except an additional gene between G and L gene (**Figure 3**). In the phylogenetic trees of L gene, the analyzed viruses are obviously divided into two groups: cytorhabdoviruses and nucleorhabdoviruses. The cytorhabdoviruses form two subgroups: one comprising viruses, including RSMV, that infect monocots, and the other representing pathogens of dicots. RSMV is transmitted by leafhoppers, where the other cytorhabdoviruses are transmitted by planthoppers, aphids or unknown vectors. GenBank accession numbers of rhabdovirus sequences used for comparisons and phylogenetic analyses are given in the Supplementary File S1.

In 2015–2016, RSMV-infected plants were found in many counties of southwestern Guangdong Province in southern China (Supplementary Figure S3). 190 suspicious samples collected from five counties were detected by RT-PCR and 146 were RSMV positive (**Table 4**). Disease incidences were generally 1 to 5%; however, in some fields, the incidence was higher than 10%, thereby seriously harming rice production (**Figures 1A–C**). RT-PCR analysis revealed that the leafhoppers R. dorsalis could acquire the virus naturally, with a viruliferous rate of 56.8% (21 of 37) in the population collected from the field with about 10% diseased plants. This leafhopper could transmit RSMV to healthy rice seedlings with a high efficiency in the artificial transmission test. All plants (5/5) were infected with the virus after 3-day inoculation by five viruliferous leafhopper nymphs. The infection rate still reached 80% (8/10) and 50% (5/10) when two and one viruliferous insects placed to each plant, respectively. All artificial-infected plants showed symptoms similar to the natural-infected ones (**Figures 1D,E**). RSMV could not be mechanically transmitted in our experiment, all 60 tested plants did not show any symptoms and were virus negative in RT-PCR detection. Other than rice, RSMV was only detected in asymptomatic crabgrass (*Digitaria sanguinalis*) collected from or adjacent to diseased rice fields, the virus was not detected in other sampled weeds.

**Table 4:** RSMV RT-PCR detection for rice samples collected from southern China in 2015 and 2016.

Year	Sampling location (County)	Sampling field	Rice cultivar	No. of samples tested	No. of RSMV positive
2015	Taiping	1	Taifengyou 55	8	6
		2	Hengfengyou 387	10	6
		3	Wuyou736	11	8
	Songgui	1	Changyou736	8	7
		2	Changyou736	11	7
		3	Guyou1263	7	4
	Luoping	1	Hengfengyou 387	8	5
		2	Yuemeizhan	9	6
	Luoding	1	Yuyou 098	10	5
		2	Guyou1263	8	4
		3	Taifengyou 55	7	4
2016	Songgui	1	Changyou736	10	10
		2	Changyou736	10	8
	Lianzhou	1	Fengtianyou116	8	6
		2	Fengtianyou116	7	6
	Taiping	1	Meiyou9822	20	20
		2	Meiyou9822	28	25
		3	Longyou766	10	9
Total				190	146

## Discussion

Rhabdoviruses are widely present in nature and have a broad host range that includes vertebrates, invertebrates and plants (Jackson et al., 2005; Kuzmin et al., 2009). More than 100 plant rhabdoviruses have been reported, but most cannot be assigned to a genus because genome sequences and clearly identified replication sites are lacking (Jackson et al., 2005). In this study, small RNA sequencing and RT-PCR were performed to acquire the complete genome of an unidentified plant virus. Based on its morphological and pathological features, and phylogenetic relationships with other plant rhabdoviruses (**Figure 5**; Supplementary Table S2), we identified the virus as a new cytorhabdovirus in family *Rhabdoviridae*, and suggested it a tentative name RSMV. To our knowledge, RSMV is the first reported cytorhabdovirus naturally infecting rice and transmitted by a leafhopper.

Under an electron microscope, rhabdovirus particles often appear enveloped and either bullet-shaped or bacilliform (Jackson et al., 2005; Kormelink et al., 2011; Dietzgen et al., 2016). In our study, electron microscopy revealed the presence of enveloped, bacilliform virions rather than bullet-shaped particles in infected rice leaf cells (**Figures 2A,B**). Additionally, some viral particles was accumulated in intracellular vesicles (**Figure 2G**). This phenomenon is probably common for the plant rhabdoviruses due to budding occurs from the inner nuclear membrane or ER membrane and cytoplasmic vesicles (Jackson et al., 2005), and was also observed in animal infecting rhabdoviruses, vesicular stomatitis indiana virus (VSIV, genus *Vesiculovirus*) (Hackett et al., 1968) and niakha virus (NIAV, genus unassigned) (Vasilakis et al., 2013) suggesting the alternative intracellular transport for virus budding in animal, but its function in plant is unclear. Furthermore, lettuce necrotic yellows virus (LNYV) particles had been observed in the perinuclear spaces with blistering on the outer nuclear membrane, and LNYV multiplication appears to occur in the nucleus at early stages (Wolanski and Chambers, 1971). In our study, examination of ultrathin sections found that some viral particles were near the nucleus and adhered to the nuclear membrane (**Figures 2C–E**), suggesting that viral RNA synthesis and/or maturation of RSMV is probably similar to LNYV at early stages of infection.

Analysis of the RSMV genome revealed that RSMV carries seven non-overlapping genes in the order 3′-N-P-P3-M-G-P6-L-5′ (**Figure 3A**). It is generally known that rhabdoviruses encoded five major structural proteins (N, P, M, G, and L protein). In this study, we confirmed that RSMV encoded N, P, M, G, and L are viral structural proteins by purified protein sequencing. We did not find other viral proteins in the purified virus preparation probably because of the sensitivity limit of the staining method in SDS-PAGE analyses. There have been researches indicating that the RYSV and sonchus yellow net virus (SNYV) viral MP and P6 protein can also be detected in their matured virions (Scholthof et al., 1994; Huang et al., 2003; Hiraguri et al., 2012). The P3 encoded by RSMV shared only 10.8% to 19.7% identities with the counterparts of other plant rhabdoviruses (**Table 2**), whether it function as a movement protein like in other virus (Walker et al., 2011; Mann and Dietzgen, 2014) need further experiment evidence. A transmembrane region is found in RSMV P6, similar to BYSMV P9 (Yan et al., 2015) and alfalfa dwarf virus (ADV) P6 (Bejerman et al., 2015), indicating it can probably be located on or transported through membrane. Its function requires further investigation, though its counterpart in RYSV has been identified as a systemic RNA silencing suppressor (Guo et al., 2013), however in LNYV the P protein serves as RNA silencing suppressor in plants (Mann et al., 2015; Bejerman et al., 2016). In addition, the seven genes are separated by conserved intergenic regions containing putative regulatory signals that have been reported in other rhabdoviruses (Jackson et al., 2005; Walker et al., 2015; Dietzgen et al., 2016). As a typical feature of all rhabdoviruses, the 3′ and 5′ end sequences of RSMV are complementary (Supplementary Figure S2) and can form a putative panhandle structure thought to be involved in genome replication (Jackson et al., 2005).

Some cytorhabdoviruses may induce host nucleus changes in the early stages of infection (Jackson et al., 2005). In the case of RSMV, the N proteins contain two predicted NLSs (**Figure 3A**), which have been identified in nucleorhabdoviruses but not cytorhabdoviruses (Jackson et al., 2005). Moreover, the ADV N and P protein complex can locate to the nucleus though the absent of NLS in them (Bejerman et al., 2015). Determining whether RSMV N protein is locate to the nuclear membranes is worthy of future study. Finally, most of the phosphorylated residues in the RSMV P protein are serine residues (29/39; **Table 1**), similar to VSV (Mondal et al., 2014).

Most rice viruses are transmitted by arthropods, so their epidemiologic and distribution dependent on their vector (Hibino, 1996). Our field investigations indicate that RSMV is now mainly distributed in southwestern Guangdong Province of China (Supplementary Figure S3), where the virus vector leafhopper *R. dorsalis* is an increasing pest in rice field (Li et al., 2015). Although RSMV disease incidence is generally 1 to 5% at present, it might become seriously epidemic in this or even larger region with the leafhopper population increase because of warm weather. We thus believe that special attention should be focused on this new pathogen to minimize the potential for future outbreaks.

## Author Contributions

GZ: Conceived and designed the experiments. XY, BC: Performed the biological experiments. JH, CL: Observed the virion morphology. TZ: Analyzed the data. All authors read and approved the final manuscript.

## Conflict of Interest Statement

The authors declare that the research was conducted in the absence of any commercial or financial relationships that could be construed as a potential conflict of interest. The reviewer HK and handling Editor declared their shared affiliation, and the handling Editor states that the process nevertheless met the standards of a fair and objective review.

## Footnotes

1 http://www.ictvonline.org/

2 http://www.ncbi.nlm.nih.gov

3 http://www.cbs.dtu.dk/services/NetNGlyc/

## References

[B1] Afonso C. L. Amarasinghe G. K. Bányai K. Bào Y. Basler C. F. Bavari S. (2016). Taxonomy of the order Mononegavirales: update 2016. *Arch. Virol.* 161 2351–2360. 10.1007/s00705-016-2880-1 27216929 PMC4947412

[B2] Ammar E. Tsai C. W. Whitfield A. E. Redinbaugh M. G. Hogenhout S. A. (2009). Cellular and molecular aspects of rhabdovirus interactions with insect and plant hosts. *Annu. Rev. Entomol.* 54 447–468. 10.1146/annurev.ento.54.110807.090454 18793103

[B3] Bejerman N. Giolitti F. de Breuil S. Trucco V. Nome C. Lenardon S. (2015). Complete genome sequence and integrated protein localization and interaction map for alfalfa dwarf virus, which combines properties of both cytoplasmic and nuclear plant rhabdoviruses. *Virology* 483 275–283. 10.1016/j.virol.2015.05.001 26004251

[B4] Bejerman N. Mann K. S. Dietzgen R. G. (2016). Alfalfa dwarf *Cytorhabdovirus* P protein is a local and systemic RNA silencing supressor which inhibits programmed RISC activity and prevents transitive amplification of RNA silencing. *Virus Res.* 224 19–28. 10.1016/j.virusres.2016.08.008 27543392

[B5] Bheemanahalli R. Sathishraj R. Tack J. Nalley L. L. Muthurajan R. Jagadish K. S. V. (2016). Temperature thresholds for spikelet sterility and associated warming impacts for sub-tropical rice. *Agric. For. Meteorol.* 221 122–130. 10.1016/j.agrformet.2016.02.003

[B6] Blom N. Gammeltoft S. Brunak S. (1999). Sequence and structure-based prediction of eukaryotic protein phosphorylation sites. *J. Mol. Biol.* 294 1351–1362. 10.1006/jmbi.1999.3310 10600390

[B7] Dietzgen R. G. Callaghan B. Wetzel T. Dale J. L. (2006). Completion of the genome sequence of *Lettuce necrotic* yellows virus, type species of the genus *Cytorhabdovirus*. *Virus Res.* 118 16–22. 10.1016/j.virusres.2005.10.024 16313992

[B8] Dietzgen R. G. Kondo H. Goodin M. M. Kurath G. Vasilakis N. (2016). The family Rhabdoviridae: mono- and bipartite negative-sense RNA viruses with diverse genome organization and common evolutionary origins. *Virus Res.* 227 158–170. 10.1016/j.virusres.2016.10.010 27773769 PMC5124403

[B9] Dietzgen R. G. Kuhn J. H. Clawson A. N. Freitas-Astúa J. Goodin M. M. Kitajima E. W. (2014). Dichorhavirus: a proposed new genus for Brevipalpus mite-transmitted, nuclear, bacilliform, bipartite, negative-strand RNA plant viruses. *Arch. Virol* 159 607–619. 10.1007/s00705-013-1834-0 24081823

[B10] Guo H. Song X. Xie C. Huo Y. Zhang F. Chen X. (2013). Rice yellow stunt rhabdovirus protein 6 suppresses systemic RNA silencing by blocking RDR6-mediated secondary siRNA synthesis. *Mol. Plant Microbe Interact.* 26 927–936. 10.1094/MPMI-02-13-0040-R 23634838

[B11] Hackett A. J. Zee Y. C. Schaffer F. L. Talens L. (1968). Electron microscopic study of the morphogenesis of *Vesicular stomatitis* virus. *J. Virol.* 2 1154–1162. 4302018 10.1128/jvi.2.10.1154-1162.1968 PMC375448

[B12] Heim F. Lot H. Delecolle B. Bassler A. Krczal G. Wetzel T. (2008). Complete nucleotide sequence of a putative new *Cytorhabdovirus* infecting lettuce. *Arch. Virol.* 153 81–92. 10.1007/s00705-007-1071-5 17943394

[B13] Hibino H. (1996). Biology and epidemiology of rice viruses. *Annu. Rev. Phytopathol.* 34 249–274. 10.1146/annurev.phyto.34.1.249 15012543

[B14] Hiraguri A. Hibino H. Hayashi T. Netsu O. Shimizu T. Uehara-Ichiki T. (2012). The movement protein encoded by gene 3 of rice transitory yellowing virus is associated with virus particles. *J. Gen. Virol.* 93 2290–2298. 10.1099/vir.0.044420-0 22815270

[B15] Huang Y. Zhao H. Luo Z. Chen X. Fang R. X. (2003). Novel structure of the genome of Rice yellow stunt virus: identification of the gene 6-encoded virion protein. *J. Gen. Virol.* 84 2259–2264. 10.1099/vir.0.19195-0 12867659

[B16] Jackson A. O. Dietzgen R. G. Goodin M. M. Bragg J. N. Deng M. (2005). Biology of plant rhabdoviruses. *Annu. Rev. Phytopathol.* 43 623–660. 10.1146/annurev.phyto.43.011205.141136 16078897

[B17] Kondo H. Maeda T. Shirako Y. Tamada T. (2006). Orchid fleck virus is a rhabdovirus with an unusual bipartite genome. *J. Gen. Virol.* 87 2413–2421. 10.1099/vir.0.81811-0 16847138

[B18] Kondo H. Maeda T. Tamada T. (2009). Identification and characterization of structural proteins of orchid fleck virus. *Arch. Virol.* 154 37–45. 10.1007/s00705-008-0268-6 19066715

[B19] Kormelink R. Garcia M. L. Goodin M. Sasaya T. Haenni A. (2011). Negative-strand RNA viruses: the plant-infecting counterparts. *Virus Res.* 162 184–202. 10.1016/j.virusres.2011.09.028 21963660

[B20] Kosugi S. Hasebe M. Tomita M. Yanagawa H. (2009). Systematic identification of cell cycle-dependent yeast nucleocytoplasmic shuttling proteins by prediction of composite motifs. *Proc. Natl. Acad. Sci. U.S.A.* 106 10171–10176. 10.1073/pnas.0900604106 19520826 PMC2695404

[B21] Kuzmin I. V. Novella I. S. Dietzgen R. G. Padhi A. Rupprecht C. E. (2009). The rhabdoviruses: biodiversity, phylogenetics, and evolution. *Infect. Genet. Evol.* 9 541–553. 10.1016/j.meegid.2009.02.005 19460320

[B22] Lamprecht R. L. Kasdorf G. G. F. Stiller M. Staples S. M. Nel L. H. Pietersen G. (2010). Soybean blotchy mosaic virus, a new *Cytorhabdovirus* found in South Africa. *Plant Dis.* 94 1348–1354. 10.1094/PDIS-09-09-0598 30743624

[B23] Li S. Hao W. Lu G. Huang J. Liu C. Zhou G. (2015). Occurrence and identification of a new vector of Rice orange leaf phytoplasma in south China. *Plant Dis.* 99 1483–1487. 10.1094/PDIS-12-14-1243-RE 30695964

[B24] Liu X. Gorovsky M. A. (1993). Mapping the 5′ and 3′ ends of Tetrahymena thermophila mRNAs using RNA ligase mediated amplification of cDNA ends (RLM-RACE). *Nucleic Acids Res.* 21 4954–4960. 10.1093/nar/21.21.4954 8177745 PMC311412

[B25] Mann K. S. Dietzgen R. G. (2014). Plant rhabdoviruses: new insights and research needs in the interplay of negative-strand RNA viruses with plant and insect hosts. *Arch. Virol.* 159 1889–1900. 10.1007/s00705-014-2029-z 24610553

[B26] Mann K. S. Johnson K. N. Dietzgen R. G. (2015). *Cytorhabdovirus* phosphoprotein shows RNA silencing suppressor activity in plants, but not in insect cells. *Virology* 476 413–418. 10.1016/j.virol.2014.12.023 25591176

[B27] Mondal A. Victor K. G. Pudupakam R. S. Lyons C. E. Wertz G. W. (2014). Newly identified phosphorylation site in the *Vesicular stomatitis* virus P protein Is Required for Viral RNA Synthesis. *J. Virol* 88 1461–1472. 10.1128/JVI.02384-13 24257610 PMC3911598

[B28] Motschulsky V. I. (1859). Homopteres. In “Insectes des Indes orientales, et de contrees analogues.” *Etud. Entomol.* 8 25–118

[B29] Ramalho T. O. Figueira A. R. Sotero A. J. Wang R. Geraldino Duarte P. S. Farman M. (2014). Characterization of Coffee ringspot virus-Lavras: a model for an emerging threat to coffee production and quality. *Virology* 46 385–396. 10.1016/j.virol.2014.07.031 25117897

[B30] Sasaya T. Ishikawa K. Koganezawa H. (2002). The nucleotide sequence of RNA1 of Lettuce big-vein virus, genus *Varicosavirus*, reveals its relation to nonsegmented negative-strand RNA viruses. *Virology* 297 289–297. 10.1006/viro.2002.1420 12083827

[B31] Sasaya T. Kusaba S. Ishikawa K. Koganezawa H. (2004). Nucleotide sequence of RNA2 of Lettuce big-vein virus and evidence for a possible transcription term ination/in itiation strategy similar to that of rhabdoviruses. *J. Gen. Virol.* 85 2709–2717. 10.1099/vir.0.80061-0 15302964

[B32] Scholthof K. G. Hillman B. I. Modrell B. Heaton L. A. Jackson A. O. (1994). Characterization and detection of sc4: a sixth gene encoded by *Sonchus* yellow net virus. *Virology* 204 279–288. 10.1006/viro.1994.1532 8091658

[B33] Tamura K. Peterson D. Peterson N. Stecher G. Nei M. Kumar S. (2011). MEGA5: molecular evolutionary genetics analysis using maximum likelihood, evolutionary distance, and maximum parsimony methods. *Mol. Biol. Evol.* 28 2731–2739. 10.1093/molbev/msr121 21546353 PMC3203626

[B34] Uehara-Ichiki T. Shiba T. Matsukura K. Ueno T. Hirae M. Sasaya T. (2013). Detection and diagnosis of rice-infecting viruses. *Front. Microbiol.* 4:289 10.3389/fmicb.2013.00289 PMC3793123 24130554

[B35] Vasilakis N. Widen S. Mayer S. V. Seymour R. Wood T. G. Popov V. (2013). Niakha virus: a novel member of the family Rhabdoviridae isolated from phlebotomine sandflies in Senegal. *Virology* 444 80–89. 10.1016/j.virol.2013.05.035 23773405 PMC3755043

[B36] Walker P. J. Dietzgen R. G. Joubert D. A. Blasdell K. R. (2011). Rhabdovirus accessory genes. *Virus Res.* 162 110–125. 10.1016/j.virusres.2011.09.004 21933691 PMC7114375

[B37] Walker P. J. Firth C. Widen S. G. Blasdell K. R. Guzman H. Wood T. G. (2015). Evolution of genome size and complexity in the Rhabdoviridae. *PLoS Pathog.* 11:e1004664 10.1371/journal.ppat.1004664 PMC4334499 25679389

[B38] Wang X. Wu Q. Ito T. Cillo F. Li W. Chen X. (2010). RNAi-mediated viral immunity requires amplification of virus-derived siRNAs in *Arabidopsis thaliana*. *Proc. Natl. Acad. Sci. U.S.A.* 107 484–489. 10.1073/pnas.0904086107 19966292 PMC2806737

[B39] Wolanski B. S. Chambers T. C. (1971). The multiplication of *Lettuce necrotic* yellows virus. *Virology* 44 582–591. 10.1016/0042-6822(71)90372-2 4109519

[B40] Yan T. Zhu J. Di D. Gao Q. Zhang Y. Zhang A. (2015). Characterization of the complete genome of Barley yellow striate mosaic virus reveals a nested gene encoding a small hydrophobic protein. *Virology* 478 112–122. 10.1016/j.virol.2014.12.042 25666524

