# Supplementary material for: Rice Stripe Mosaic Virus, a Novel Cytorhabdovirus Infecting Rice via Leafhopper Transmission
Source: Front Microbiol. 2017 Jan 4;7:2140. doi: 10.3389/fmicb.2016.02140 (PMC5210121; doi:10.3389/fmicb.2016.02140)
Supplement: Supplementary file 1 [file Data_Sheet_1.docx]

**Supplementary Material**

**S1 | GenBank accession numbers used in this study.**

GenBank accession numbers for genome sequences of other rhabdoviruses used in this study are as follows: *Alfalfa dwarf cytorhabdovirus* (ADV, KP205452), *Barley yellow striate mosaic cytorhabdovirus* (BYSMV, KM213865), Colocasia bobone disease-associated virus (CBDaV, KT381973), *Coffee ringspot dichorhavirus* (CoRSV, KF812525, KF812526), *Lettuce big-vein associated varicosavirus* (LBVaV, NC011558, NC011568), *Datura yellow vein nucleorhabdovirus* (DYVV, NC028231), *Eggplant mottled dwarf nucleorhabdovirus* (EMDV, NC025389), *Lettuce necrotic yellows cytorhabdovirus* (LNYV, NC007642), *Lettuce yellow mottle cytorhabdovirus* (LYMoV, NC011532), *Maize ﬁne streak nucleorhabdovirus* (MFSV, NC005974), *Maize Iranian mosaic nucleorhabdovirus* (MIMV, NC011542), *Maize mosaic nucleorhabdovirus* (MMV, NC005975), *Northern cereal mosaic cytorhabdovirus* (NCMV, NC002251), *Orchid fleck dichorhavirus* (OFV, NC009608, NC009609), Persimmon virus A (PeVA, AB735628), *Potato yellow dwarf nucleorhabdovirus* (PYDV, GU734660), *Rice yellow stunt nucleorhabdovirus*  (RYSV, NC003746), *Sonchus yellow net nucleorhabdovirus* (SYNV, NC001615), *Taro vein chlorosis nucleorhabdovirus* (TaVCV, NC006942).

**TABLE S1 | Primer sequences used in this study.**

| Primer name | Primer sequence (5'-3') | Purpose | Primer position |
| --- | --- | --- | --- |
| Adaptor-R | GGAACTGACACAGAGTGATCAGCTGCAT | viral RNA 3' terminus | 203-223  112-131 |
| N-F1 | GACCTCTTCTCTAGCTTGTAG |  |  |
| N-F2 | AACCAGGCTCAGCTTCTCCT |  |  |
| Adaptor-F | ATGCAGCTGATCACTCTGTGTCAGTTCC | viral cDNA 3' terminus |  |
| L-R1 | TCTCTAAGGTGGCCTCAATGT |  | 12458-12478 |
| L-R2 | CTGATTGGCAGTATCCGAGTC |  | 12663-12683 |
| N-F1 | TACGGATAATACTGGCAGAAGC | gap between N and G | 1435-1456 |
| G-R1 | GTCGTGCTCCTTAGACCTCTT |  | 3464-3484 |
| G-F1 | ACAATGGTTGAGACCGTGAAAG | gap between G and L | 5776-5797 |
| L-R1 | ATCCAGCCTAGTGATTTCATCC |  | 6425-6446 |
| RSMV-F | TGCACAGACGTTAGTGAGTTAC | RSMV detection | 7164-7185 |
| RSMV-R | TCCGTCTTTCATAGCCTTCAG |  | 7666-7686 |
| F-A | GAAAAACGAGACCGCCAAGTCT | RSMV genome confirmation | 64-85 |
| R-A | AGGCACAAGATCACAGACGAT |  | 2862-2882 |
| F-B | TCAGTTACTACCTGTGTGGCA |  | 2809-2829 |
| R-B | TAGCACGTCTCCATAGACCA |  | 6303-6322 |
| F-C | ACCTTGATGACGGTGGTCTAT |  | 6290-6310 |
| R-C | TCTTCCTGATTACGCTCACGA |  | 10472-10492 |

lue-R250 () in 12% SDS-PAGE gels followed by staining with Coomassie brillint
3URE 5S1 columnsNAl siRNA al siRNA in major 21 nt lengths samples. NA is more than antigenome-derived siRNA, F = forward; R = reverse

**TABLE S2 | Comparison (identities, %) of nucleotide and amino acid sequences of RSMV ORFs with equivalent regions of other plant rhabdoviruses.**

| Virus | Nucleo capsid | | Phosphor protein | | P3 protein | | Matrix protein | | Glycol protein | | P6 protein | | L polymerase | |
| --- | --- | --- | --- | --- | --- | --- | --- | --- | --- | --- | --- | --- | --- | --- |
|  | NT | AA | NT | AA | NT | AA | NT | AA | NT | AA | NT | AA | NT | AA |
| ADV | 44.8 | 18.6 | 45.3 | 16.8 | 27.6 | 13.5 | 28.2 | 12.0 | 46.1 | 20.0 | 46.3 | 20.3 | 48.4 | 27.4 |
| BYSMV | 50.8 | 32.4 | 47.6 | 21.0 | 31.6 | 14.5 | 28.2 | 11.4 | 47.2 | 21.9 | 48.4 | 18.0 | 52.1 | 39.4 |
| CBDaV | 48.3 | 33.0 | 44.2 | 16.8 | 33.5 | 12.9 | 29.1 | 13.5 | 49.1 | 19.6 | - | - | 52.5 | 38.7 |
| LYMoV | 45.6 | 21.4 | 46.4 | 14.0 | 30.9 | 13.5 | 31.2 | 10.3 | 47.0 | 19.2 | - | - | 48.9 | 27.3 |
| LNYV | 46.3 | 17.7 | 48.3 | 16.0 | 30.8 | 13.5 | 27.7 | 10.3 | 47.3 | 18.3 | - | - | 47.1 | 27.4 |
| NCMV | 49.6 | 31.2 | 45.4 | 18.1 | 30.1 | 13.3 | 29.5 | 13.7 | 49.6 | 22.5 | - | - | 51.9 | 38.6 |
| PeVA | 45.1 | 18.4 | 44.3 | 15.0 | 30.3 | 12.4 | 28.6 | 12.6 | 45.6 | 20.6 | - | - | 47.3 | 26.5 |
| DYVV | 45.5 | 17.3 | 47.9 | 14.9 | 27.9 | 12.4 | 31.4 | 13.1 | 48.1 | 16.8 | - | - | 47.8 | 21.7 |
| EMDV | 44.6 | 13.8 | 49.0 | 12.9 | 30.4 | 13.5 | 29.9 | 12.0 | 45.7 | 15.0 | - | - | 47.3 | 23.7 |
| MFSV | 47.6 | 17.9 | 46.9 | 16.8 | 29.2 | 15.2 | 27.0 | 12.0 | 48.1 | 14.4 | - | - | 47.6 | 22.4 |
| MIMV | 44.9 | 12.4 | 43.4 | 17.3 | 30.8 | 13.5 | 29.0 | 14.3 | 46.9 | 16.1 | - | - | 48.4 | 24.0 |
| MMV | 44.8 | 20.3 | 46.5 | 17.0 | 29.0 | 11.2 | 28.8 | 13.7 | 46.2 | 16.8 | - | - | 48.3 | 23.1 |
| PYDV | 43.8 | 21.5 | 45.6 | 16.4 | 28.4 | 12.9 | 27.7 | 11.4 | 45.3 | 14.7 | - | - | 47.8 | 22.2 |
| RYSV | 44.7 | 12.6 | 44.6 | 16.4 | 26.9 | 12.9 | 25.2 | 12.0 | 47.0 | 19.8 | 42.3 | 34.3 | 47.9 | 22.7 |
| SYNV | 45.1 | 11.6 | 46.0 | 14.4 | 31.4 | 14.0 | 28.2 | 12.0 | 46.5 | 16.1 | - | - | 46.1 | 20.9 |
| TaVCV | 44.0 | 13.3 | 46.4 | 17.3 | 32.2 | 13.5 | 29.0 | 12.0 | 46.4 | 18.1 | - | - | 47.5 | 24.1 |
| CoRSV | 32.7 | 13.2 | 34.8 | 13.4 | 43.7 | 15.2 | 35.0 | 13.3 | 40.3 | 14.5 | - | - | 42.6 | 23.4 |
| OFV | 35.2 | 12.2 | 36.0 | 12.6 | 40.7 | 19.7 | 36.6 | 15.2 | 39.3 | 14.8 | - | - | 41.0 | 24.1 |
| LBVaV | 44.3 | 15.8 | 40.1 | 12.9 | 40.0 | 10.8 | 37.2 | 9.3 | 36.0 | 11.0 | 41.5 | 9.8 | 39.4 | 22.1 |

NT = nucleotide; AA = amino acid.


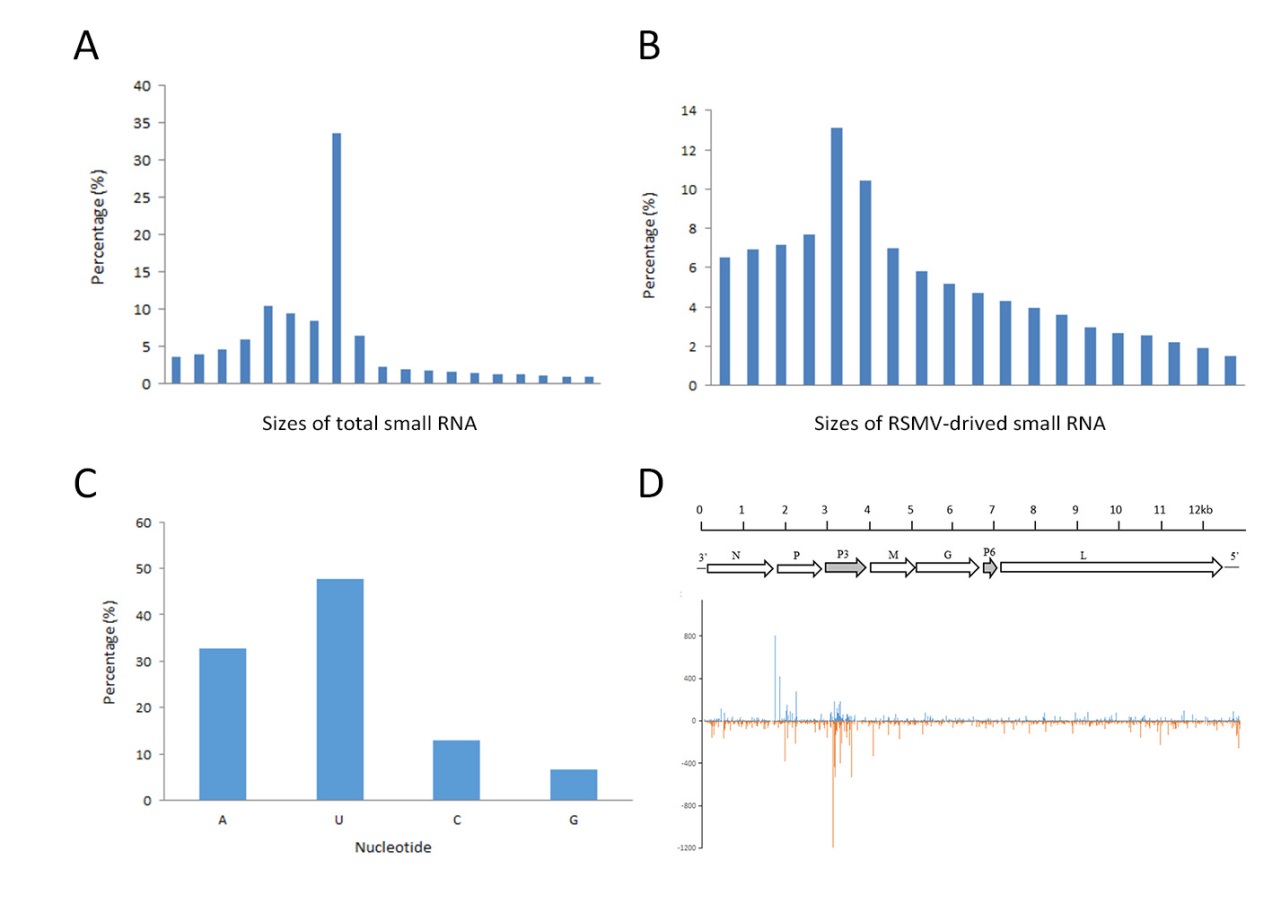


**FIGURE S1 | Profile of the small RNA in RSMV-infected rice leaves. (A)** Size distribution of total small RNA. (B) Size distribution of RSMV-derived small RNA. (C) Relative frequency of the 5′-terninat nucleotide of 21 nt viral major small RNA. (D) Distribution of viral small RNA along the antigenomic RNA (up, blue columns) and genomic RNA (down, red columns).


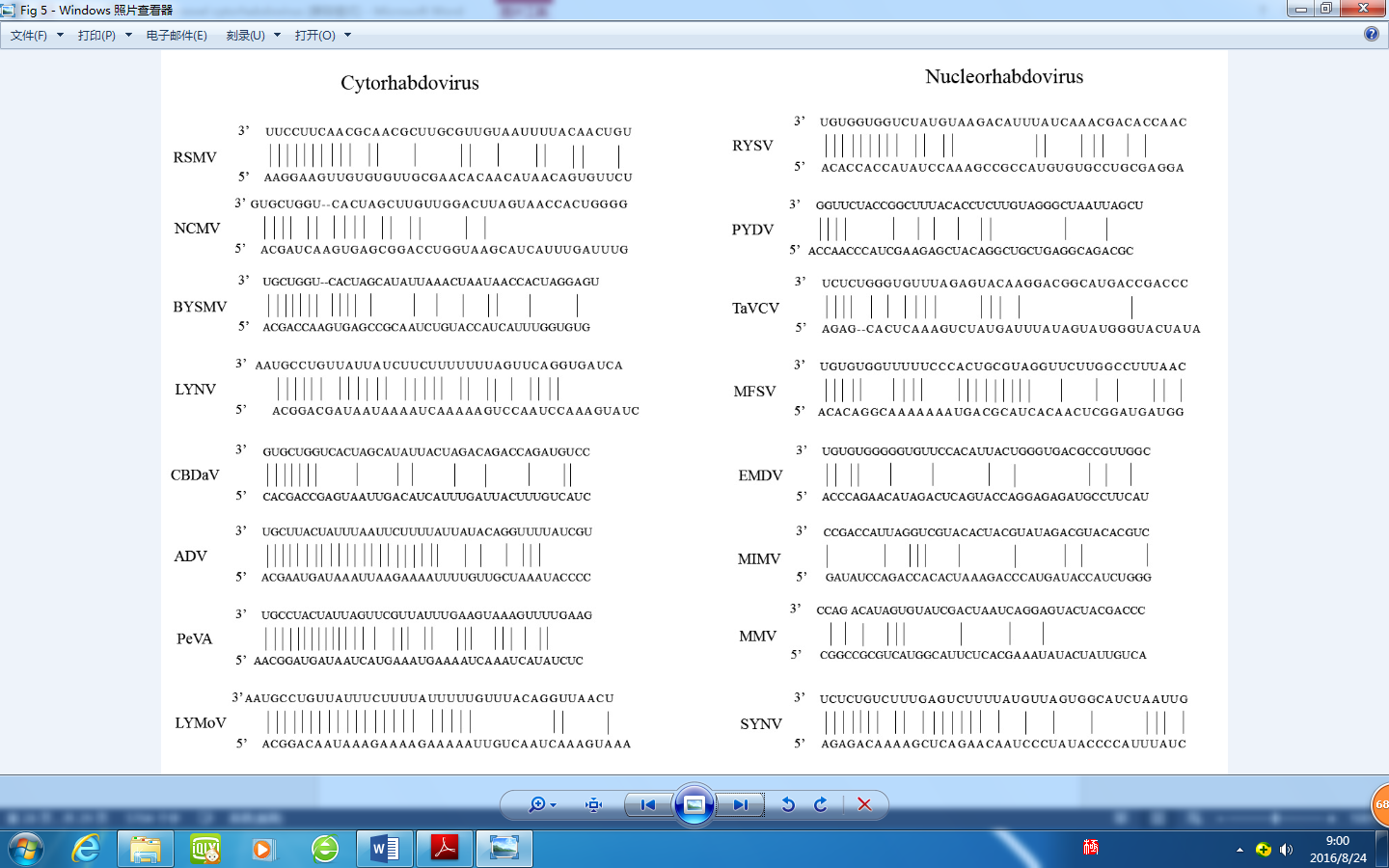


**FIGURE S2 | Characteristics of non-coding terminal sequences of RSMV and other plant rhabdovirus genomes.** Sequence complementarity of genomic 3′ and 5′ terminals of RSMV and plant-infecting rhabdoviruses (*Cytorhabdovirus* and *Nucleorhabdovirus*) is shown.


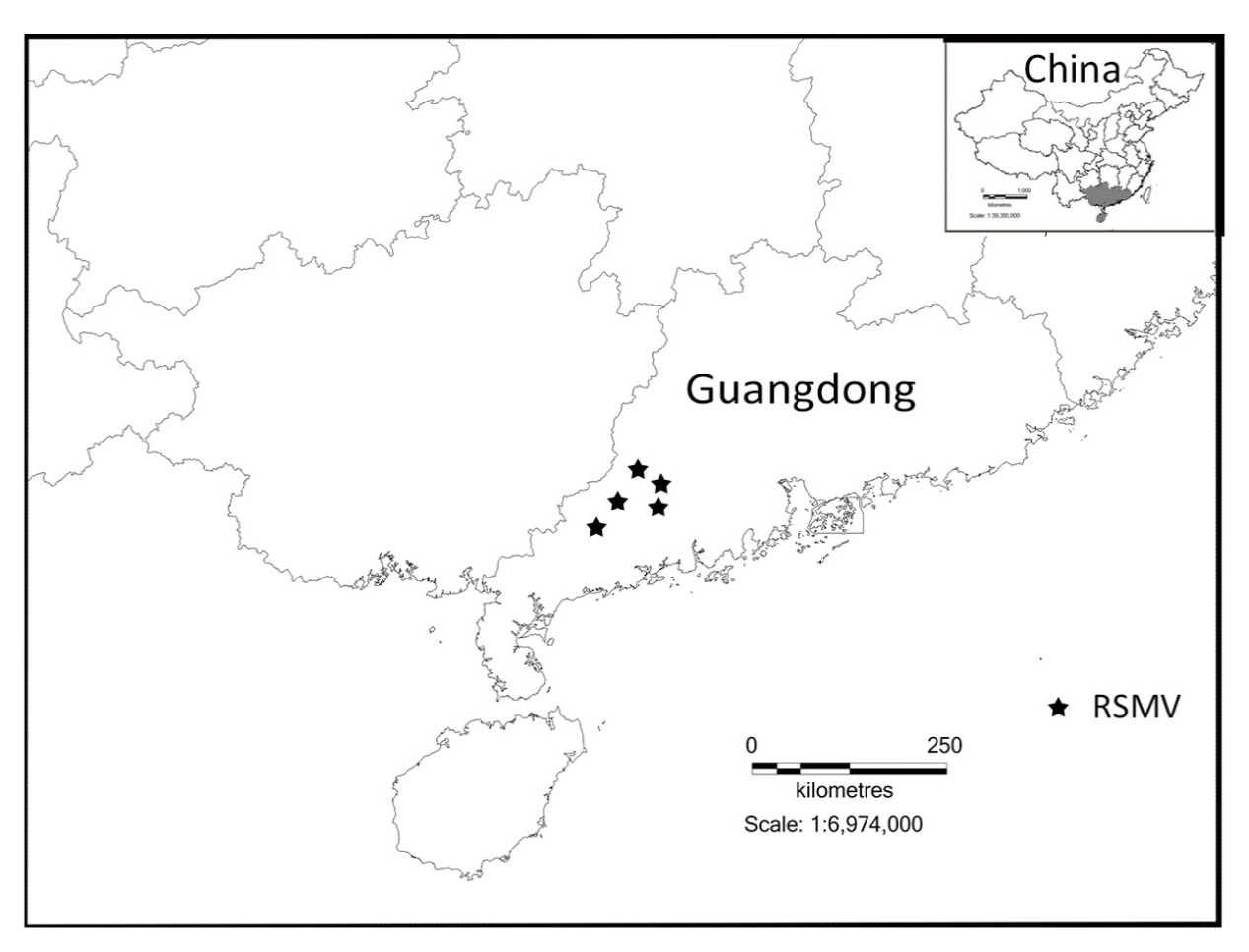


**FIGURE S3 | Geographic distribution of RSMV in year 2015 and 2016 revealed by this study.**
